# P2X_7_ receptor-nitric oxide interaction mediates apoptosis in mouse immortalized mesangial cells exposed to high glucose

**DOI:** 10.1590/2175-8239-JBN-2021-0086

**Published:** 2021-10-15

**Authors:** Thamires de Oliveira Fernandes, Adelson Marçal Rodrigues, Giovana Rita Punaro, Deyse Yorgos de Lima, Elisa Mieko Suemitsu Higa

**Affiliations:** 1 Universidade Federal de São Paulo, Divisão de Nefrologia, São Paulo, SP, Brasil.; 2 Universidade Federal de São Paulo, Depardamento de Medicina, São Paulo, SP, Brasil.; 3 Universidade Federal de São Paulo, Laboratório de Óxido Nítrico e Estresse Oxidativo, São Paulo, SP, Brasil.; 4 Universidade Federal de São Paulo, Divisão de Emergência, São Paulo, SP, Brasil.

**Keywords:** Mesangial Cells, Glucose, Apoptosis, Nitric Oxide Synthases, Receptors, Purinergic, Nitric Oxide, Células Mesangiais, Glicose, Apoptose, Óxido Nítrico Sintase, Receptor Purinérgico, Óxido Nítrico

## Abstract

**Introduction:**

Diabetes mellitus (DM) is a chronic disease characterized by hyperglycemia that leads to diabetic nephropathy (DN). We showed that P2X_7_, a purinergic receptor, was highly expressed in DM; however, when oxidative stress was controlled, renal NO recovered, and the activation of this receptor remained significantly reduced. The aim of this study was to assess the influence of NO on the P2X_7_ and apoptosis in mouse immortalized mesangial cells (MiMC) cultured in high glucose (HG) medium.

**Methods:**

MiMCs were cultured with DMEM and exposed to normal glucose (NG), mannitol (MA), or HG. Cell viability was assessed by an automated counter. Supernatants were collected for NO quantification, and proteins were extracted for analysis of NO synthases (iNOS and eNOS), caspase-3, and P2X_7_.

**Results:**

Cell viability remained above 90% in all groups. There was a significant increase in the proliferation of cells in HG compared to MA and NG. NO, iNOS, caspase-3, and P2X_7_ were significantly increased in HG compared to NG and MA, with no changes in eNOS. We observed that there was a strong and significant correlation between P2X_7_ and NO.

**Discussion:**

The main finding was that the production of NO by iNOS was positively correlated with the increase of P2X_7_ in MCs under HG conditions, showing that there is a common stimulus between them and that NO interacts with the P2X_7_ pathway, contributing to apoptosis in experimental DM. These findings could be relevant to studies of therapeutic targets for the prevention and/or treatment of hyperglycemia-induced kidney damage to delay DN progression.

## Resumo

**Introdução:**

Diabetes mellitus (DM) é uma doença crônica caracterizada por hiperglicemia levando à nefropatia diabética (ND). Mostramos que P2X_7_, um receptor purinérgico, foi altamente expresso na DM; entretanto, quando o estresse oxidativo foi controlado, o NO renal recuperou-se, e a ativação deste receptor permaneceu significativamente reduzida. Este estudo objetivou avaliar a influência do NO no P2X_7_ e a apoptose em células mesangiais imortalizadas de camundongos (CMiC) cultivadas em meio de glicose elevada (GE).

**Métodos:**

CMiCs foram cultivadas em meio DMEM e expostas à glicose normal (GN), manitol (MA), ou GE. A viabilidade celular foi avaliada por contador automático. Sobrenadantes foram coletados para quantificação de NO, e foram extraídas proteínas para análise de NO sintases (iNOS e eNOS), caspase-3, e P2X_7_.

**Resultados:**

A viabilidade celular permaneceu acima de 90% em todos os grupos. Houve aumento significativo na proliferação de células na GE comparado com MA e GN. NO, iNOS, caspase-3 e P2X_7_ foram significativamente aumentados na GE comparados com GN e MA, sem alterações na eNOS. Observamos que houve correlação forte e significativa entre P2X_7_ e NO.

**Discussão:**

O principal achado foi que a produção de NO pela iNOS foi positivamente correlacionada com aumento de P2X_7_ em CMs sob condições de GE, mostrando que existe um estímulo comum entre eles e que o NO interage com a via do P2X_7_, contribuindo para apoptose na DM experimental. Estes achados podem ser relevantes para estudos de alvos terapêuticos para a prevenção e/ou tratamento de danos renais induzidos por hiperglicemia para retardar a progressão da ND.

## Introduction

Diabetic nephropathy (DN) affects approximately 40% of patients with diabetes and involves a number of alterations in glomerular filtration and in the mechanisms of tubular reabsorption, as well as morphologic changes in the renal tissue, resulting in chronic renal failure^1^. A study showed that the main change occurs at the glomerular level, resulting in expansion of the mesangial extracellular matrix (ECM), hypertrophy, and proliferation of mesangial cells (MCs), playing an important role in the development of DN^2^. The insulin effect on MCs is not yet clear; insulin has an essential role in glucose metabolism, in addition to having other controversial effects, such as its relationship with fibrosis^3^ and maintainer of MC function^4^.

MCs have many functions, including the control of glomerular filtration and blood flow regulation of the glomeruli. They can also produce NO and inflammatory mediators, including cytokines^5^. In addition, MCs have several receptors for a wide variety of hormones and growth factors, developing multiple physiological functions in glomeruli^6^^-^ 8; in pathological conditions, the release of several factors in the body can trigger the expression of other receptors in the MC, including purinergic receptors^9^.

P2 receptors have been divided into two large families: P2X and P2Y. The P2X family can be expressed in every organism and consists of seven subunits (P2X1-7). Generally, it acts as an ion key-lock channel, and its signals are formed by two transmembrane domains separated by an extracellular domain, with two cytoplasmic extremities (N and C)^10^.

The P2X_7_ receptor, unlike others, needs high concentrations of ATP to be activated. The release of ATP in several cells is a physiological response to mechanical stress, inflammation, hypoxia, or certain agonists^10^. The stimulation of the P2X_7_ receptor results in the production of cytokines such as interleukin 1β (IL-1β) and tumor necrosis factor alpha (TNF-α), as well as in the synthesis of reactive oxygen species (ROS)^11^.

The P2X_7_ receptor is expressed at low levels in the kidney under normal conditions^12^, but in an ex vivo study in our laboratory, we demonstrated that the P2X_7_ receptor was highly expressed in diabetic animals, and when the oxidative stress of those animals was controlled, there was a recovery of renal NO with a significant reduction in the activation of this receptor^13^, but we did not know if there was any relationship between P2X_7_ and NO.

Considering that MCs are the cells most affected by hyperglycemia in the kidneys, the aim of the present study was to investigate the possible interaction between P2X_7_ receptor and NO bioavailability in immortalized mouse mesangial cells in an environment that mimics diabetes mellitus.

## Material and Methods

### Mesangial cell culture

MiMC purchased from American Type Culture Collection (ATCC - CRL 1927) was provided by the Nephrology Division - Federal University of Sao Paulo (UNIFESP, SP, Brazil). The cells were grown and kept in a 95% air and 5% CO_2_ humidified environment at 37ºC in Dulbecco's modified Eagle's medium (DMEM) and F12 (3:1) (Vitrocell, Sao Paulo, Brazil) containing 5% fetal bovine serum (FBS). The medium was replaced every 48 h. All experiments were performed with cells between the 11^th^ and 16^th^ passages. The ideal time (72 h) for treatment in our study was determined according to the time response curve of NO production of MiMC exposed to HG, considering that in this period, there was higher NO bioavailability. Recent studies have used a dose of 30 mM glucose to mimic DM^14^. The protocol was approved by the Ethics Committee in Research of the Federal University of Sao Paulo, under the number 7215080115.

Cells were cultured in medium with 1% FBS at approximately 50% semiconfluence, which is the ideal cell density to avoid overgrowth while allowing an increase in cell quality and avoiding any stress or production of deleterious agents that could affect the experiment. The experimental groups were: NG, which was cultured in DMEM containing a standard concentration of 5.5 mM D-glucose; HG, cultured with DMEM containing D-glucose at a final concentration of 30 mM; and the osmolarity control, which was cultured in DMEM supplemented with MA at a final concentration of 30 mM.

### Cell viability and proliferation

MiMCs were cultured with 5% FBS in 12-well plates at a concentration of 5x10^4^ cells/mL per well. At semiconfluence (50%), the cells were exposed to a medium containing 1% FBS in conditions of NG, MA or HG. Then, the cells were trypsinized and centrifuged, the supernatant was discarded, and the cells were resuspended in 1 mL fresh medium. Cell viability was assessed using trypan blue (0.4%)^15^. The cells were counted using an automated cell counter (Countess, Invitrogen, Carlstadt, USA).

### Nitric oxide measurement

Twelve-well plates with a concentration of 5x10^4^ cells/mL MiMC per well were treated according to their respective groups. After the treatment, the supernatant was collected and stored in a freezer at -20°C. The NO levels in the supernatant were measured by chemiluminescence using the Nitric Oxide Analyzer (NOA 280, Sievers Instruments Inc, CO, USA), which is a high-sensitivity detector for measuring NO (~1 pmol) based on the gas-phase chemiluminescent reaction between NO and ozone^13^. The sample is injected into the equipment, and through a reaction with vanadium chloride, the stable metabolites nitrite and nitrate are reconverted into NO, which is then measured. This technique is considered the gold standard for NO analysis. The values were corrected by the protein concentration using the bicinchoninic acid (BCA) protein assay (Sigma-Aldrich Chemical CO, MO, USA).

### Western blot analysis

The cells were cultured in Petri dishes at a concentration of 1x10^6^ cells/mL and treated with NG, HG, or MA. Then, the cells were lysed with RIPA buffer containing 50 mM tris-HCl, 150 mM NaCl, 0.25% acid deoxycholic, 1% nonidet P-40, 0.1% SDS, 1 mM EDTA, and protease inhibitor (Millipore, Sao Paulo, Brazil). The protein was concentrated with ultra-filter 0.5 mL, with pore size or nominal molecular weight limit of 50 kDa (Millipore, Sao Paulo, Brazil), and determined by BCA protein assay (Sigma-Aldrich Chemical CO, MO, USA). A total of 40 µg protein concentrate was applied to a 10% polyacrylamide gel and transferred to a nitrocellulose membrane. Nonspecific binding was blocked with 10% nonfat dry milk in a pH 7.5 TBS-T buffer followed by washing in the same buffer at room temperature. The membranes were then incubated overnight at 4ºC with primary and secondary antibodies against eNOS (1:200 and 1:1000), iNOS (1:200 and 1:1000), caspase-3 (1:500 and 1:5000), P2X_7_ (1:1000 and 1:2000), and actin (1:5000 and 1:10000) (Santa Cruz Biotechnology Inc., CA, USA). The specific protein bands were visualized using Immobilon western chemiluminescent HRP substrate (Millipore Corporation MA, USA), and analysis was performed using *ImageJ software (*US National Institutes of Health, MD, USA).

### Statistical analysis

The results are expressed as the mean and standard error of the median (SEM). The differences among the three groups were examined for statistical significance using one-way analysis of variance (ANOVA) followed by Newman-Keuls Multiple Comparison post-test for parametric data (NO, viable and total cells, iNOS, eNOS, and caspase-3) or Kruskal-Wallis followed by Dunn's Multiple Comparison post-test for non-parametric data (dead cells and P2X_7_). Values were considered statistically significant when p<0.05. The correlation between P2X_7_ and NO was analyzed by Pearson's test. Statistical analysis was performed in the program GraphPad Prism 5.0 (GraphPad Software Inc., USA).

## Results

### Cell viability and proliferation and no production

**Figure 1:**
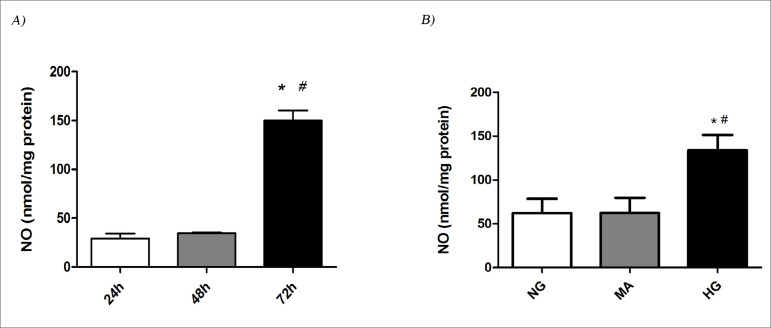
Time response of NO production after 24, 48 or 72 h of MiMC incubation in HG and NO bioavailability of MiMC after 72 h in NG, MA or HG media. NO: nitric oxide; NG: normal glucose (5.5 mM); MA: mannitol (30 mM); HG: high glucose (30 mM). MiMC: mouse immortalized mesangial cell. Values are expressed as mean and SEM. n=5-7 per group. One-way ANOVA with Newman-Keuls post-test; p<0.05: ^&^vs 24 h; ^ɣ^vs 48h; *vs NG; ^#^vs MA.

The chosen time for HG treatment was 72 h because at this time, the highest NO production occurred (150.2 ± 10.2) compared to 24 or 48 h (29.2 ± 4.9 and 34.6 ± 0.7, respectively; p<0.0001). NO production was higher after 72 h (133.7 ± 17.6) of treatment with HG than after 24 h (62.4 ± 17) or 48 h (62.2 ± 16.1), p<0.05, Figure 1.

After 72 h of HG treatment, the viable, nonviable, and total number of cells was significantly increased compared to the NG or MA groups, and the percentage of viable cells in each group was approximately 94-96% (Table 1).

**Table 1:** Viability and proliferation of MiMC in the NG, MA and HG groups after 72h

	NG	MA	HG
Viable (10^6^ cells/mL)	1.40 ± 0.8	1.20 ± 0.1	2.30 ± 0.1*^#^
Dead (10^6^ cells/mL)	0.06 ± 1.0	0.06 ± 1.0	0.13 ± 0.9*^#^
Total (10^6^ cells/mL)	1.46 ± 0.1	1.26 ± 0.1	2.43 ± 0.1*^#^
Viable cells (%)	96	95	95

### Nos analysis

**Figure 2:**
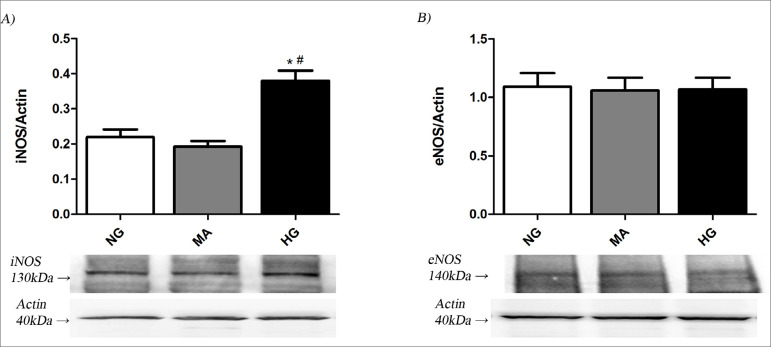
A) iNOS and B) eNOS after 72 h in HG conditions. iNOS: inducible nitric oxide synthase; eNOS: endothelial nitric oxide synthase; NG: normal glucose (5.5 mM); MA: mannitol (30 mM); HG: high glucose (30 mM), n=12 per group. One-way ANOVA with Newman-Keuls post-test; p<0.05: *vs NG; ^#^vs MA

The iNOS in MiMC after 72 h was significantly higher in HG (0.38 ± 0.02) than in NG or MA (0.22 ± 0.02; 0.19 ± 0.01, respectively; p<0.05) (Figure 2A). The eNOS showed no difference among groups in the same period (Figure 2B).

### Analysis of caspase-3 and p2x_7_ receptor in mimc

**Figure 3:**
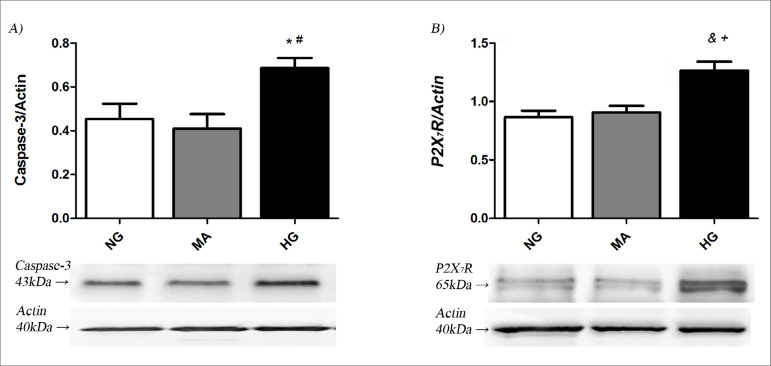
A) Caspase-3 and B) P2X7R after 72 h in HG conditions. NG: normal glucose (5.5 mM); MA: mannitol (30 mM); HG: high glucose (30 mM), n=12 per group. One-way ANOVA with Newman-Keuls post test or Kruskal-Wallis test with Dunn's post-test; p<0.05: *vs NG; ^#^vs MA.

The caspase-3 protein content, a predictor of apoptosis, was increased in the HG group after 72 h (0.68 ± 0.04) compared to the NG or MA groups (0.45 ± 0.07; 0.41 ± 0.06, respectively; p<0.05), as shown in Figure 3A. P2X_7_ receptor was significantly increased in the HG group after 72 h of treatment (1.26 ± 0.07) compared to the other groups, NG (0.87 ± 0.05) and MA (0.90 ± 0.05), Figure 3B.

### P2x_7_ receptor and no correlation

**Figure 4:**
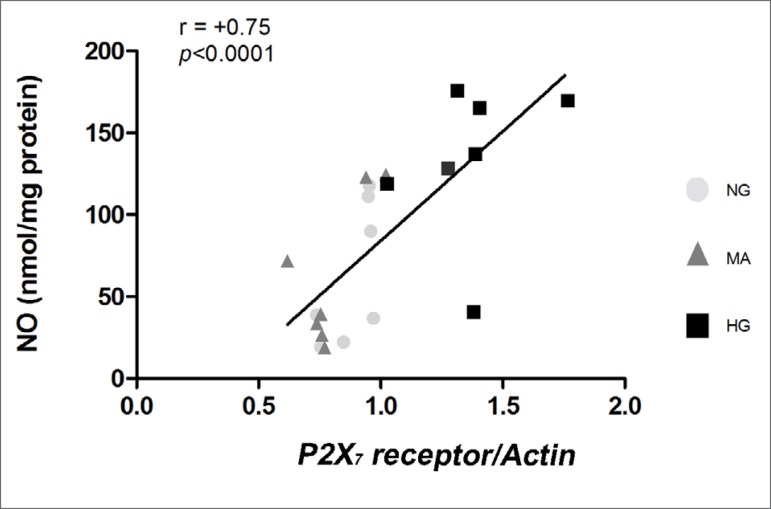
Correlation between P2X_7_ receptor and NO in MiMC after 72 h in HG conditions. NG: normal glucose (5.5 mM); MA: manitol (30 mM); HG: high glucose (30 mM), n=7 per group. Pearson's test, coefficient r = 0.75, p<0.0001

A significant, strong and positive correlation was found between P2X_7_ receptor and NO levels in all groups (p<0.0001, r = +0.75), as shown in Figure 4.

## Discussion

In the present study, MiMC showed increased proliferation and mortality when treated with HG compared to cells exposed to NG; we believe that cell death was due to apoptosis, since caspase-3 was elevated in this group. However, these changes did not affect the viability of these cells. In the HG group, an increase in the P2X_7_ receptor and iNOS isoform was also observed.

A major finding of our study was that the increased P2X_7_ receptor was strongly correlated with NO bioavailability in cells cultured with HG. We suspect that this increase in NO was due to iNOS, since there was no difference in eNOS among groups. The finding is important because it might explain one of the mechanisms by which hyperglycemia induces an increase in the P2X_7_ receptor. At the same time, it would cause an imbalance of the nitrosative/oxidative system, due to the combination of NO with the superoxide anion, leading to the production of peroxynitrite, a potent cytotoxic agent 16, resulting in greater glomerular damage during the progression of DM.

Several studies have shown that HG can stimulate the MC proliferation by several mechanisms, including the accumulation of ROS^(17, 18)^. A recent study showed that MC proliferation and intense production of ECM are key factors in the development of DN^19^. This study partially corroborates our data, as the cells treated with high glucose medium presented intense proliferation.

MCs have several functions under physiological conditions, such as moderating ECM synthesis, endocytosis, glomerular hemodynamics, permeability ENT#091;20ENT#093;, and NO synthesis ENT#091;21ENT#093;. Exposure of these cells to HG medium promotes the appearance of oxidative stress, and consequently, an increase in NO production via iNOS through activation of the PI3K / Akt pathway 22 ^,^ 23

These findings are consistent with our study in that we found an increase in iNOS synthesis, and we hypothesize that this is a critical factor for the increase in NO production as the other isoform under study, eNOS, showed no difference among groups. The endothelial isoform of NO synthase has an important role in the systemic and renal hemodynamic maintenance^24^^,^ 25.

Solini et al. (2005)^26^ showed that MCs exposed to high glucose increased ATP production in relation to normal glucose-treated cells^26^. Another study demonstrated that the release of ATP by the activation of PI3K, Rho kinase, or increased calcium concentration is common in medium with HG and occurs in different cell types^27^. The events that result in increased extracellular ATP concentration can lead to a cascade of actions such as stimulation of purinergic signaling 28. Previous studies by Vonend et al. (2004)^12^ showed that the P2X_7_ receptor in MCs is produced at low levels under normal conditions, but in an inflammatory environment, its synthesis increases and requires large amounts and continuous stimulation of extracellular ATP for its production^29^^,^ 30. These data agree with our study, since P2X_7_ receptor was increased significantly in MCs after 72 h of exposure to HG medium.

P2X_7_ is a cell death receptor involved in apoptosis and necrosis, leading to pore formation and rupture of the plasma membrane^31^. However, some authors have shown that this receptor also participates in the proliferation of lymphoid cells^32^, microglia^33^ and glomerular cells, including MCs^12^. Therefore, in our study, we believe that this receptor plays an important role in proliferation and apoptosis of cells exposed to high glucose.

P2X_7_ can also participate in the production of proinflammatory cytokines, particularly IL-1β, IL-18, and TNF-α, which can result in the activation of iNOS and increase of the production of superoxide anion and NO levels^11^^,^ 34 ^,^ 35. Since 1996, Park et al.^36^ and cols had already demonstrated that there was a relationship between the increase in the intracellular calcium concentration and iNOS expression, and this interaction occurs by the activation of purinergic receptors through ATP in different cell types^(37, 38)^. These studies corroborate our findings once iNOS was increased in the HG group, which was probably due to the release of proinflammatory cytokines mediated by P2X_7_ receptor.

In our research, we showed that the expression of P2X_7_ receptor was significantly and strongly associated with the increase in NO levels in the cells treated with HG, probably due to the increased production of iNOS. It is known that both iNOS and P2X_7_ expression are dependent on inflammatory agents^(39, 40)^, which indicates that there is a gap in this respect, and more studies are necessary for a better characterization and understanding of the inflammatory profile of MCs in HG conditions, but we believe that both have a common trigger in addition to stimulation by hyperglycemia.

These results can lead to a better understanding of how HG affect MCs and induce a high mortality index, as was shown in the present study. With HG, there is a reduction of some functions, which could be the key elements for DN progression. Thus, P2X_7_ receptor has become an important protagonist in therapies for diseases with high levels of oxidative stress and cell death, such as DN. A diabetic rat model showed that inactivation of P2X_7_ by its antagonist improved kidney injury via reduction of proinflammatory macrophages^41^.

A study carried out in our team showed that the P2X_7_ receptor was associated with redox imbalance in response to oxidative stress control. Later we showed that P2X_7_ was expressed in small amounts during the weeks of diabetes and had a peak expression in the 6^th^ week, resulting in high lipid peroxidation and reduced NO levels in the kidney^42^. The findings of this study corroborate the initial phase of the above-mentioned study, in which the slightly elevated P2X_7_ levels were accompanied by higher levels of renal NO in these diabetic kidneys, since the incubation time of MC in the HG medium was 72 h, i.e., less than one week of DM.

The silencing of P2X_7_ receptor demonstrated its kidney deleterious effect as its low expression improved kidney function and balanced oxidative and nitrosative profiles, demonstrating that inhibiting P2X_7_ can benefit the kidneys and slow DN progression^43^. In addition, we found that calcium entrance by P2X_7_ was intense when DM did not have adjuvant therapy. High levels of free calcium in the cytoplasm trigger apoptotic mechanisms manifested by mitochondrial stress, cytochrome c release, and caspase 3 formation, demonstrating that P2X_7_ extremely elevates intracellular calcium^43^. It was also observed that the partial absence of P2X_7_ modulates the renin-angiotensin system and increases NO levels 44.

The limitations of our study were the lack of nitrosative stress markers such as 3-nitrotyrosine or peroxynitrite analyses, assessment of other elements of the apoptotic cascade, and calcium levels measurement.

**Figure 5:**
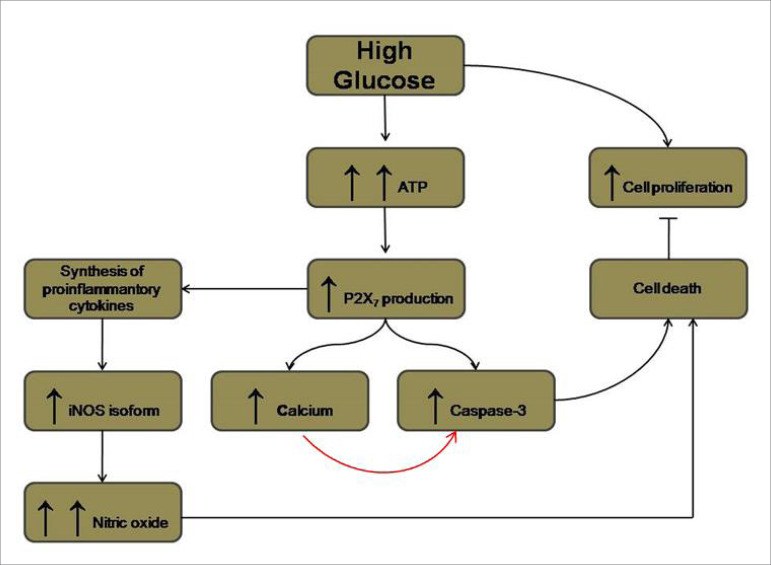
Organogram - Schematic shows how high glucose likely acts on mesangial cells. ATP - adenosine triphosphate; iNOS - inducible nitric oxide synthase; ↑ - increase.

To summarize our findings, we present how we think works the hypothetical mechanism by which HG initially leads to an increase in P2X_7_ production, triggering a cascade of events and resulting in MC proliferation and/or apoptosis (organogram Figure 5.).
